# Genomic Responses to Abnormal Gene Dosage: The X Chromosome Improved on a Common Strategy

**DOI:** 10.1371/journal.pbio.1000318

**Published:** 2010-02-23

**Authors:** Xinxian Deng, Christine M. Disteche

**Affiliations:** 1 Department of Pathology, University of Washington, Seattle, Washington, United States of America; 2 Department of Medicine, University of Washington, Seattle, Washington, United States of America

## Abstract

This new primer, which discusses a study by Zhang et al., provides an overview of the process by which chromosomes achieve dose compensation and the mechanisms underlying this phenomenon in *Drosophila* S2 cells.

Mechanisms to guard genomic integrity are critical to ensuring the welfare and survival of an organism. Disruptions of genomic integrity can result in aneuploidy, a large-scale genomic imbalance caused by either extra or missing whole chromosomes (chromosomal aneuploidy) or chromosome segments (segmental aneuploidy). A change in dosage of a single gene may not compromise the well-being of an organism, but the combined altered dosage of many genes due to aneuploidy disturbs the overall balance of gene expression networks, resulting in decreased fitness and mortality [1],[2]. Chromosomal aneuploidy is a common cause of birth defects—Down syndrome is caused by an extra copy of Chromosome 21, and Turner syndrome by a single copy of the X chromosome in females. Furthermore, methods that detect segmental aneuploidy have uncovered small deletions or duplications of the genome in association with many disorders, such as mental retardation. Chromosomal and segmental aneuploidies are also frequent in cancer cells in which changes in copy number paradoxically increase cell fitness but are unfavorable to survival of the organism. A fundamental issue in biology and medicine is to understand the effects of aneuploidy on gene expression and the mechanisms that alleviate aneuploidy-induced imbalance of the genome.

Chromosomal aneuploidy is caused by non-disjunction of chromosomes in meiosis or mitosis, while segmental aneuploidy involves breakage and ligation of DNA. In contrast, the sex chromosomes provide an example of a naturally occurring “aneuploidy” caused by the evolution of a specific set of chromosomes for sex determination that often differ in their copy number between males and females. For example, in mammals and in flies, females have two X chromosomes and males have one X chromosome and a Y chromosome, resulting in X monosomy in males. How does a cell or an organism respond to such different types of aneuploidy, abnormal or natural? It turns out that the overall expression level of a given gene is not necessarily in direct relation to the copy number. Unique strategies have evolved to deal with abnormal gene dosage to alleviate the effects of aneuploidy by dampening changes in expression levels. What's more, the X chromosome has evolved sophisticated mechanisms to achieve complete dosage compensation, not surprisingly, since the copy number difference between males and females has been evolving for a long time.

## Gene Expression Responses to Altered Dosage in Aneuploidy

**Figure 1:**
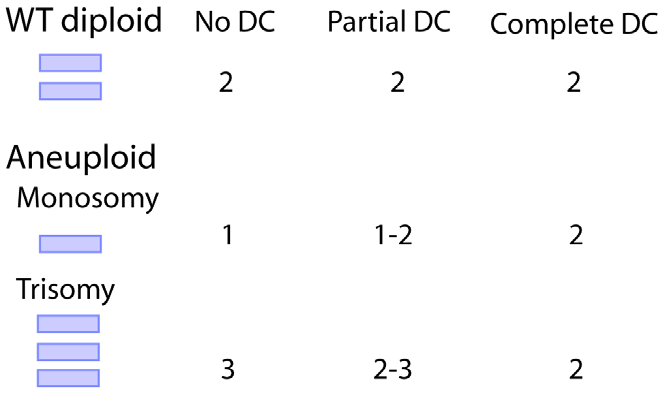
Expression levels change in response to altered gene dose in aneuploidy. The transcript output from a given pair of chromosomes in normal WT diploid cells is set as a value of 2. In case of aneuploidy (monosomy or trisomy), the amount of transcript would be strictly correlated with gene dose in the absence of a dosage compensation mechanism (No DC). In the presence of partial DC, the expression level per copy would be partially increased in monosomy or partially decreased in trisomy, relative to the diploid level. In the presence of complete DC, expression levels would be adjusted so that the amount of transcripts is the same in monosomic or trisomic cells compared to diploid cells.

There are two main outcomes from altered gene dosage in aneuploidy in terms of transcript levels—either levels directly correlate with gene dosage (primary dosage effect) or they are unchanged/partially changed with gene dosage (complete or partial dosage compensation) [3]. In the first scenario, a reduction of the normal gene dosage in a wild-type (WT) diploid cell from a symbolic dose value of 2 to a value of 1 after a chromosomal loss or deletion would produce half as many gene products, while an increase in gene dosage from 2 to 3, due to a chromosomal gain or duplication, would produce 1.5-fold more products (Figure 1). In the second scenario, the amount of products from altered gene dosage would either equal or nearly equal that in WT cells, due to complete or partial compensation (Figure 1).

Gene expression analyses of aneuploid cells or tissues in human, mouse, fly, yeast, and plant provide examples of both primary dosage effects and dosage compensation. Hence, changes in expression levels due to chromosomal aneuploidy do not affect all genes in the same manner. For example, in Down syndrome, 29% of transcripts from human Chromosome 21 are overexpressed (22% in proportion to gene dosage and 7% with higher expression), while the rest of genes are either partially compensated (56%) or highly variable among individuals (15%) [4]. Interestingly, dosage-sensitive genes, such as genes encoding transcription factors or ribosomal proteins, are more likely to be compensated to avoid harmful network imbalances [1],[5]. This basal dynamic dosage compensation could be due to buffering, feedback regulation, or both, depending on the gene and the organism [4],[6]–[9]. Buffering, a passive process of absorption of gene dose perturbations, is due to inherent non-linear properties of the transcription system. In contrast, feedback regulation is an active mechanism that detects abnormal transcript abundance and adjusts transcription levels.

## Sex Chromosome-Specific Dosage Compensation

Sex chromosome-specific dosage compensation evolved in response to the dose imbalance between autosomes and sex chromosomes in the heterogametic sex due to the different number of sex chromosomes between the sexes—for example, a single X chromosome and a gene-poor Y chromosome in males and two X chromosomes in females. Compensatory mechanisms that restore balance both between the sex chromosomes and autosomes and between the sexes vary among species [10],[11]. In *Drosophila melanogaster* (fruit fly), expression from the single X chromosome is specifically enhanced two-fold in males, while no such upregulation occurs in females. X upregulation also occurs in *Caenorhabditis elegans* (round worm) and in mammals but in both sexes [6],[12]. Silencing of one X chromosome in mammalian females and partial repression of both X chromosomes in *C. elegans* hermaphrodites have been adapted to avoid too high an expression level of X-linked genes in the homogametic sex. A unified theme in these diverse mechanisms of sex chromosome dosage compensation is coordinated upregulation of most X-linked genes approximately two-fold to balance their expression with that of autosomal genes present in two copies. This process utilizes both genetic and epigenetic mechanisms to increase expression of an X-linked gene once it has lost its Y-linked partner during evolution.

While the mechanisms of X upregulation in mammals and worms are not clear, *Drosophila* X upregulation is mediated by the male-specific lethal (MSL) complex [10],[13]. The MSL complex binds hundreds of sites along the male X chromosome and modifies its chromatin structure by MOF (males absent on the first)–mediated acetylation of histone H4 at lysine 16. Other histone modifications and chromatin-associated proteins, including both activating and silencing factors, are also involved in the two-fold upregulation of the *Drosophila* male X chromosome [14]. How these modifications coordinately work to fine-tune a doubling of gene expression is still not well understood. Moreover, the basal dynamic dosage compensation response observed in studies of autosomal aneuploidy could also play a role in *Drosophila* X upregulation [3]. An important question is how much this basal response to the onset of aneuploidy contributes to sex chromosome–specific dosage compensation.

## Fine-Tuning of the *Drosophila* X Chromosome Adds a Special Layer of Regulation above a Genome-Wide Response to Aneuploidy

In this issue of *PLoS Biology*, Zhang et al. [15] report that the exquisitely precise X chromosome upregulation in *Drosophila* utilizes both a basal response to aneuploidy and an X chromosome–specific mechanism. The beauty of their experimental system, the S2 cell line derived from a male fly, is that it has a defined genome with numerous segmental aneuploid regions, both autosomal and X-linked. Thus, genomic responses to aneuploidy could be queried both on autosomes and on the X chromosome, the latter being associated to the MSL complex. Using second-generation DNA- and RNA-sequencing, the authors carefully examined the relationship between gene copy number and gene expression in S2 cells before and after induced depletion of the MSL complex. By this approach the effects of the MSL complex on the genome have effectively been separated from those triggered by a basal response to aneuploidy.

What Zhang et al. have found is that partial dosage compensation of both autosomal and X-linked regions occurs even in the absence of the MSL complex. This provides strong evidence that basal dosage compensation mediated by buffering and feedback pathways allows dosage compensation across the whole genome. In the presence of the MSL complex, X-linked genes, but not autosomal genes, become subject to an additional level of regulation, which increases expression independent of gene copy or expression levels. This feed-forward regulation of the X chromosome by the MSL complex ensures a highly stable doubling of expression specific to this chromosome. Note that this feed-forward regulation results in precise dosage compensation only when X dose is half of the autosome dose, while insufficient or excessive X-linked gene expression occurs at lower or higher X dose. Excessive X expression has also been reported when ectopic expression of MSL2 is induced in *Drosophila* females, which leads to binding of the MSL complex to both X chromosomes and lethality [16].

**Figure 2:**
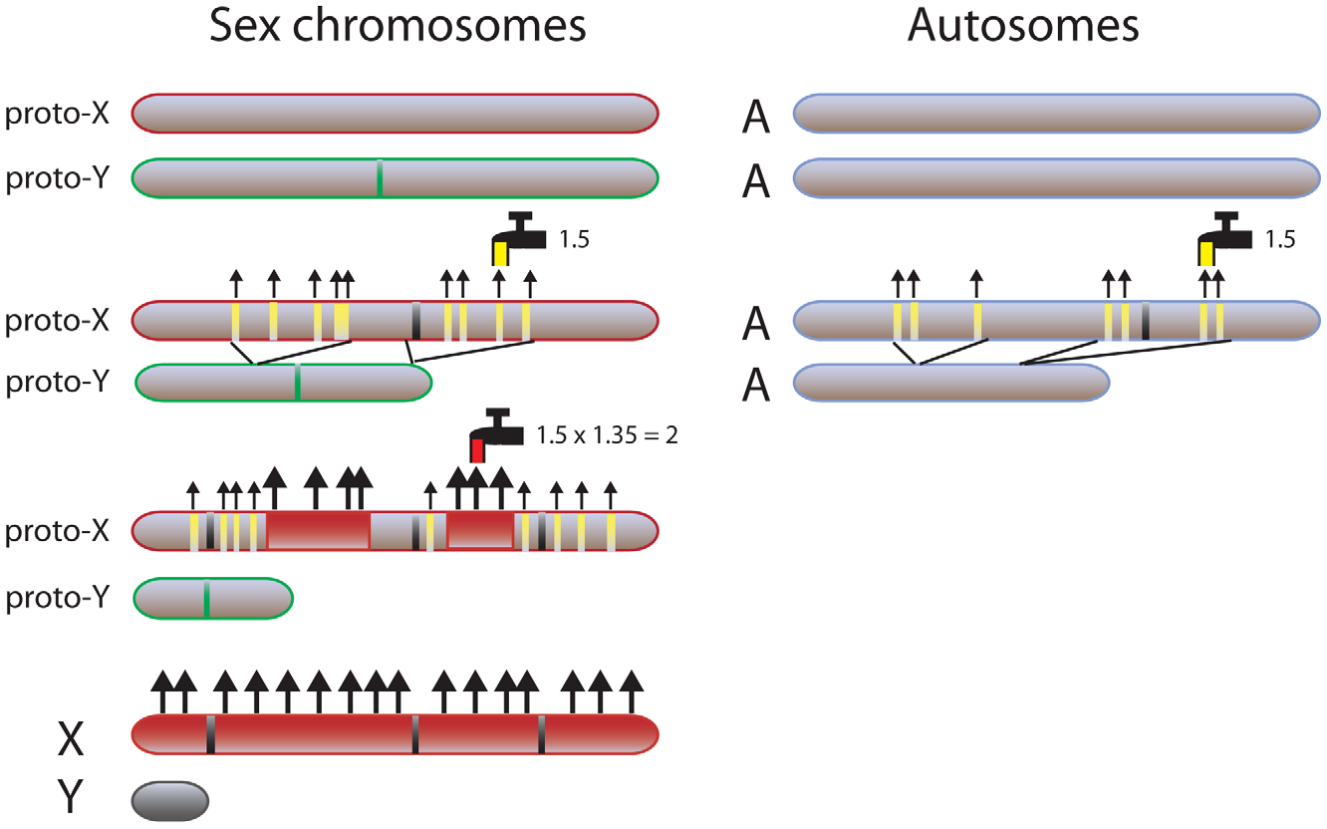
Evolutionary model of sex chromosome dosage compensation compared to the basal compensation response of an autosome after a deletion. After the proto-Y chromosome evolved a gene with a male-determining function (green bar), it became subject to gradual gene loss on a gene-by-gene or segment-by-segment basis due to lack of recombination between the proto-sex chromosomes. If the lost region on the proto-Y chromosome contained dosage sensitive genes such as those that encode transcriptional factors (yellow bars), this would have triggered a basal dosage compensation response (yellow faucet) on the proto-X chromosome and led to a partial (1.5-fold) increase of expression (small arrows). The same basal dosage compensation process would also modify a deleted region on an autosome (A) in an abnormal cell. Dosage-insensitive genes (black bars) may escape this process. When broader regions were lost on the proto-Y chromosome, the collective imbalance effects of multiple aneuploid genes would have become highly deleterious and the increased load of aneuploidy could have stressed the basal mechanism of dosage compensation. Survival was achieved by recruiting regulatory complexes such as the MSL complex (red faucet) to aneuploid X segments (red regions), to further increase gene expression (big arrows) and rescue the X monosomy. This feed-forward sex chromosome–specific regulation would provide 1.35-fold increase in expression, which together with the basal dosage compensation (1.5-fold increase) would achieve the approximate two-fold upregulation of most genes on the present day X chromosome. In contrast, large-scale deleterious autosomal aneuploidy would be lost due to lack of a specific sex-driven compensatory mechanism.

The new findings by Zhang et al. implicate two levels of regulation of the X chromosome: one basal mechanism that can regulate both the X and the autosomes in the event of aneuploidy; and a second feed-forward mechanism specific to the X and regulated by the MSL complex to ensure doubling of X-linked gene expression (Figure 2). The new study proposes that the basal compensation mechanism provides a 1.5-fold increase in gene expression and the feed-forward mechanism, another 1.35-fold, resulting in a precise two-fold increase in expression of X-linked genes. The specificity of the MSL-mediated mechanism to double X-linked gene expression is ensured by the existence of DNA sequence motifs specifically enriched on the X chromosome to recruit the MSL complex only to this chromosome [14]. Autosomal aneuploidy would only trigger a response of the basal dosage compensation pathway, which would result in a 1.5-fold increase in expression of genes located within a monosomic segment (Figure 2). It should be noted that since gene expression levels were measured relative to whole genome expression (due to normalization) a fold change in expression of genes in an aneuploid segment could also be interpreted as a fold change in expression of the rest of the genome.

How did such a precise mechanism evolve to ensure appropriate expression of sex-linked genes? The feed-forward process mediated by the MSL complex is a highly stable epigenetic modification selected and maintained during the evolution of heteromorphic sex chromosomes (Figure 2). Heteromorphic sex chromosomes have arisen from an ancestral pair of autosomes, following inhibition of recombination between the proto-Y chromosome that carries the male determinant and its counterpart, the proto-X chromosome [13]. Gradual loss of Y-linked genes due to lack of recombination could have happened gene-by-gene or on a chromosomal segment-by-segment basis. The human Y chromosome apparently evolved by a series of large inversions leading to a rapid loss of large chromosomal segments [17]. If the lost Y segments contained dosage sensitive genes, this would probably have triggered a basal dosage compensation response as observed in autosomal aneuploidy (Figure 2). However, this type of dosage compensation is dynamic and incomplete, as it is probably mediated by buffering or feedback mechanisms. An organism might tolerate partial imbalances as long as those were small, but extensive gene loss from the Y chromosome would eventually have caused a deleterious collective imbalance for multiple X-linked genes. A progressive increase in the size of aneuploid X regions could have reached a threshold of unsustainable stress on the basal dosage compensation process. To relieve this stress and survive X aneuploidy, specific mechanisms of dosage compensations targeted to the X chromosome would be desirable. Such mechanisms probably derived by recruiting pre-existing regulatory complexes, for example in the making of the MSL complex in *Drosophila*. Indeed, one of the components of this complex is MOF, a histone acetyltransferase also involved in autosomal gene regulation [10],[13]. Homologues of *Drosophila* MSL proteins also exist in other organisms where they are involved in gene regulation and DNA replication and repair but do not appear to associate with the X chromosome, suggesting that the components of X chromosome–specific complexes may differ between organisms [18].

In conclusion, two mechanisms apparently collaborate to achieve the approximate two-fold upregulation of the *Drosophila* X chromosome: a dynamic basal dosage compensation mechanism probably mediated by buffering and feedback processes; and a feed-forward, sex chromosome–specific regulation chiefly mediated by the MSL complex. In mammals, upregulation of the X chromosome may also result from a combination of more than one mechanism, some applicable to aneuploidy that may arise anywhere in the genome and others that evolved to control the X chromosome. High X-linked gene expression in mammalian cells with two active X chromosomes—undifferentiated female embryonic stem (ES) cells [19] and human triploid cells [20]—suggests that X upregulation does not default in these cells. Thus, in mammals, X upregulation may also be mediated by a highly stable feed-forward mechanism that acts on top of a basal aneuploidy response. In contrast, the sex chromosomes of birds and silkworms, ZZ in males and ZW in females, seem to lack a precise dosage compensation mechanism of the Z chromosome, possibly due to the absence of a feed-forward process [21],[22]. The Z chromosome could have a biased paucity of dosage-sensitive regulatory genes, or else selection for sexual traits may have favored the retention of gene expression imbalances between males and females. Male and female mammals display significant expression differences of a subset of genes that escape X inactivation and thus have higher expression in females [23]. Whether such genes play a role in female-specific functions is unknown. Future work to uncover the actual molecular mechanisms underlying the basal and feed-forward regulatory pathways should help to fully understand the role of these processes in different organisms, both in response to the acute onset of aneuploidy and in evolution of sex-specific traits. Loss or dysregulation of dosage compensation mechanisms could be important in birth defects and in diseases, such as cancer, where aneuploidy is common; exploring approaches to enhance dosage compensation may be useful to relieve aneuploidy-related diseases.

## Abbreviations

ES: embryonic stem
MOF: males absent on the first
MSL: male-specific lethal
WT: wild-type
